# Ontology Design Patterns for bio-ontologies: a case study on the Cell Cycle Ontology

**DOI:** 10.1186/1471-2105-9-S5-S1

**Published:** 2008-04-29

**Authors:** Mikel Egaña Aranguren, Erick Antezana, Martin Kuiper, Robert Stevens

**Affiliations:** 1 School of Computer Science, University of Manchester, Oxford Road, M13 9PL Manchester, UK; 2 Department of Plant Systems Biology, VIB, Technologiepark 927, 9052 Gent, Belgium; 3 Department of Molecular Genetics, Ghent University, Technologiepark 927, 9052 Gent, Belgium

## Abstract

**Background:**

Bio-ontologies are key elements of knowledge management in bioinformatics. Rich and rigorous bio-ontologies should represent biological knowledge with high fidelity and robustness. The richness in bio-ontologies is a prior condition for diverse and efficient reasoning, and hence querying and hypothesis validation. Rigour allows a more consistent maintenance. Modelling such bio-ontologies is, however, a difficult task for bio-ontologists, because the necessary richness and rigour is difficult to achieve without extensive training.

**Results:**

Analogous to design patterns in software engineering, Ontology Design Patterns are solutions to typical modelling problems that bio-ontologists can use when building bio-ontologies. They offer a means of creating rich and rigorous bio-ontologies with reduced effort. The concept of Ontology Design Patterns is described and documentation and application methodologies for Ontology Design Patterns are presented. Some real-world use cases of Ontology Design Patterns are provided and tested in the Cell Cycle Ontology. Ontology Design Patterns, including those tested in the Cell Cycle Ontology, can be explored in the Ontology Design Patterns public catalogue that has been created based on the documentation system presented ().

**Conclusions:**

Ontology Design Patterns provide a method for rich and rigorous modelling in bio-ontologies. They also offer advantages at different development levels (such as design, implementation and communication) enabling, if used, a more modular, well-founded and richer representation of the biological knowledge. This representation will produce a more efficient knowledge management in the long term.

## Background

Ontologies are engineering artefacts that can formally represent the concepts and their relationships within a given knowledge domain. They can provide a computationally processable conceptual representation of our current understanding of reality, as described within the information we hold. Bio-ontologies (ontologies that represent concepts from life sciences and, in particular, from molecular biology) are becoming increasingly important [1]. Bio-ontologies play a central role in bioinformatics: they act as knowledge bases, database integrators, shared vocabularies, and more [1]. Many bio-ontologies are available through the Open Biomedical Ontologies (OBO) project [2], with the Gene Ontology (GO) [3] being the most important example.

Bio-ontologies are implemented in different Knowledge Representation (KR) languages, differing in properties that can be described along the following axes:

• Syntax: what constitutes a well formed statement.

• Semantics: what well formed statements mean, often defined as the set of concrete situations (models) that are consistent with a sentence or set of sentences.

• Expressiveness: ability of the language to distinguish different kinds of concrete situations—something that can be called “precision”.

• Reasoning: answering some semantic based query, such as determining if one statement follows from another. Reasoning is performed by a program called a “reasoner”.

The most used KR languages in bioinformatics are OBO [4] and/or OWL [5]. OWL has three sub-languages, depending on the expressivity: OWL-Lite, OWL-DL and OWL-Full. OWL-Full is the most expressive type, and reasoning results are not warranted. The expressiveness of a KR language can be exploited to produce rich bio-ontologies, that is, bio-ontologies that represent the knowledge most accurately, precisely and comprehensively, with the highest possible resolution. Rich bio-ontologies are amenable to more diverse interactions with biologists, for example when querying. A rich bio-ontology can also facilitate more interesting reasoning, for example to obtain new hypotheses from biological knowledge. Presently, however, bio-ontologies mainly tend to be *lean*, as opposed to *rich*, due to the gap between the potential of KR techniques and their actual implementation in bio-ontologies. Most bio-ontologies do not come close to using all the expressiveness of the selected KR language [6], even if that language has limitations in its ability to fully describe the biological domain knowledge [7]. As a result, only a limited part of the domain knowledge is captured.

Another problem with current bio-ontologies is the lack of rigour (use of strict, explicit and well defined semantics). Rigour ensures a sound structure and hence a more robust development and maintenance over time. Despite efforts to improve the rigour of some bio-ontologies [8-10], rigorous modelling is not general practice within bio-ontologies.

The modelling effort required for obtaining a rich and rigorous bio-ontology is usually too demanding for many bio-ontologists, as they are usually biologists with a limited training in either ontology development or the KR language used for the ontology's representation. If, however, we are to improve the knowledge management in bioinformatics and move from lean to rich bio-ontologies, the bio-ontologies must be built by expert biologists who really know the vital subtleties of the knowledge domain. This tension between modelling best practice and modelling skills [11] is a fundamental barrier for progress in bio-ontologies, as the bio-ontologists only rarely use the whole power of KR languages.

One way to help bio-ontologists to model in a rich and rigorous manner is to provide them with “cookbook recipes” named Ontology Design Patterns (ODPs). ODPs are a development paradigm analogous to Software Design Patterns (SDPs) [12], widely used in OOP. A SDP is a proven solution to a known modelling problem that repeatedly appears when designing different software systems. Moreover, SDPs offer an “off the shelf” solution for the programmer: for example, in the case of the Model-View-Controller SDP a method for implementing graphical interfaces is provided. We propose that ODPs offer similar advantages to the bio-ontologists.

Structures similar to ODPs have already been used in ontologies and appear in the literature. There are, however, still open issues, such as documentation, representation, application methods, detection of application targets, *etc*. In addition, whole areas of biological knowledge lack ODPs. The work presented here begins to tackle those issues by providing a definition and classification of ODPs, methodologies for spotting application targets for ODPs in bio-ontologies, methodologies for applying ODPs, a documentation system and an ODPs public catalogue [13]. Some examples of ODPs that have been used on the Cell Cycle Ontology (CCO) are presented as use cases (Sequence ODP [14] and Upper Level Ontology ODP [15]).

## Results

### Definition and classification of ODPs

ODPs are solutions to modelling problems that help the bio-ontologist to better use the expressivity and rigour of the KR language of choice. ODPs are examples of solutions, rather than abstract solutions that are instantiated in different systems, unlike SDPs. Thus, the bio-ontologist uses the ODP as a guide and is able to recreate the ODP in the concrete bio-ontology that it is being built.

**Figure 1:**
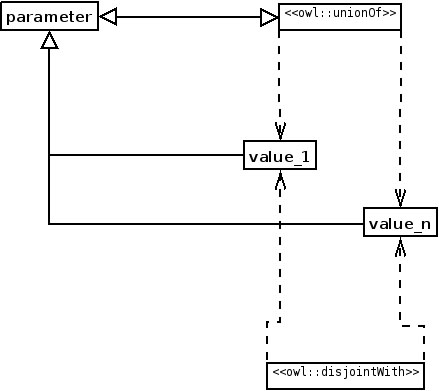
**Structure of the Value Partition ODP, in UML.** The covering axiom (the class **parameter** is equivalent to the union of classes **value_1** to **value_n**) ensures that when a new class is added, it is added as a subclass of the values; thus, no new values can be added.

**Figure 2:**
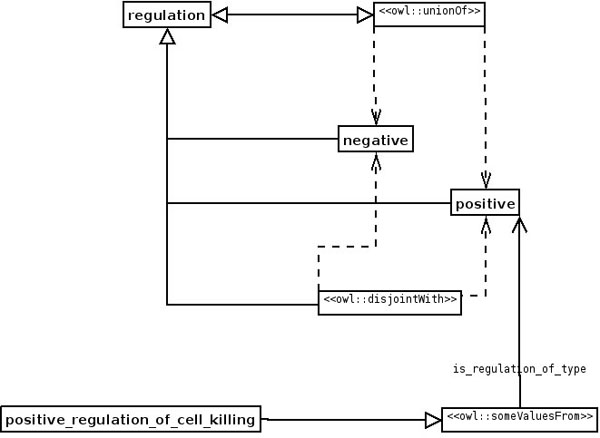
**UML diagram of the application of the Value Partition ODP.** The Value Partition is used to model biological regulation, which can only be either positive or negative, by applying the ODP described in Figure 1 to an actual bio-ontology.

ODPs are used as samples of knowledge. For example, a bio-ontologist may want to model biological regulation, which can only be positive or negative. What constructs does OWL, for example, offer to create such a model of regulation and how can the bio-ontologist combine them? The answer is to use the Value Partition ODP [16] as a sample (Figure 1; for the UML to OWL mapping used in Figures 1, 2, 6, 7, 8, 9 and 10, see Figure 4). The Value Partition ODP consists of a covering axiom and disjoint axioms that allow the values a parameter may take to be captured precisely. For example, a person can only be tall or short, but not both; the Value Partition ODP also makes the property by which an object ‘bears’ the value functional—so an object only has that property once. Since regulation can only be positive or negative (in this view of the world), this Value Partition ODP should be used (Figure 2).

ODPs are in principle abstract and implementation independent. We focus on OWL to provide a framework for direct implementation, adequate expressivity and ease of sharing. ODPs could be described in a more abstract formalism (such as First Order Logic) but that would decrease usability. ODPs can be classified according to their complexity, granularity, usability, popularity, *etc*. Here, we classify them according to the way they are used:

**Figure 3:**
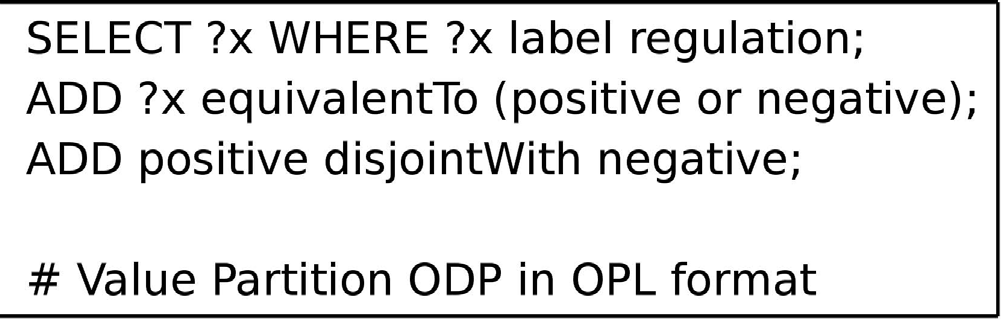
**Extract of an OPL flat file, to be processed by the OPL program**. The program reads the flat file and performs the actions in the ontology. The statements end with ; and the comments (starting with #) are not processed, **?x** is equivalent to “any class”. The statements to be processed in this example are a **SELECT** statement followed by two **ADD** statements. When parsing, the program will select any class that has the value **regulation** in its **label** annotation property. The **ADD** statements are applied to any matching classes obtained from the **SELECT** statement. It will add two axioms to any matching class: the first axiom sets the matching class to be equivalent to the union of the (already existing) classes **positive** and **negative**. The second statement makes those classes disjoint. The resulting structure is the recreation of the Value Partition ODP.

**Figure 4:**
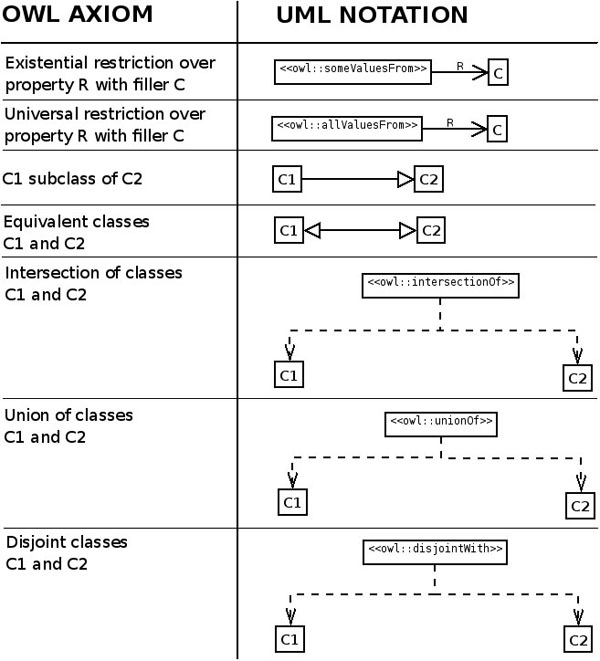
**Simple mapping of OWL to UML.** Not all the possible OWL axioms are included. R: property, C: class.

**Figure 5:**
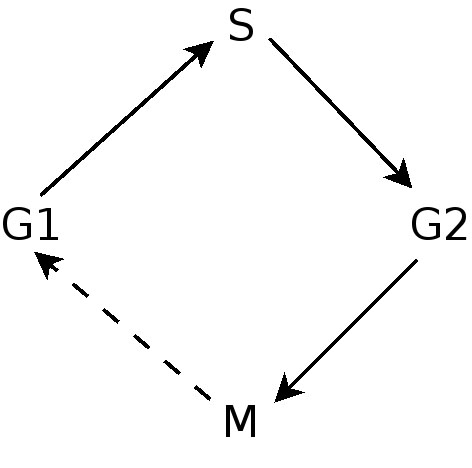
**Simplified model of cell cycle.** This simplified view of the cell cycle is assumed when modelling it using the Sequence ODP. The model, however, suffices to represent many facts about the cell cycle.

**Figure 6:**
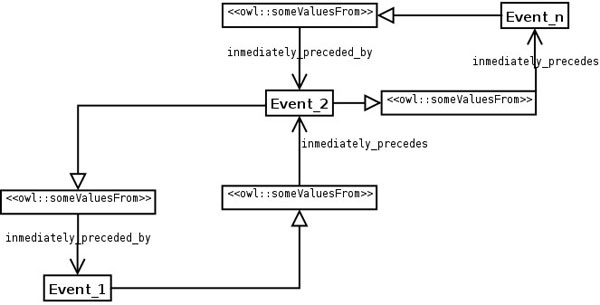
UML diagram of the Sequence ODP

**Figure 7:**
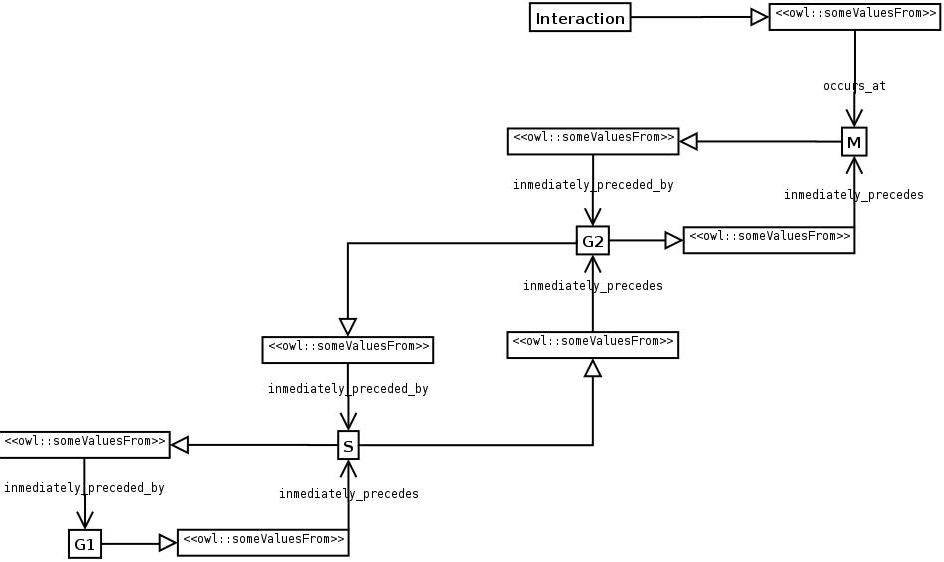
**UML diagram of the application of the Sequence ODP to CCO**. The cell cycle is defined as a sequence of phases that happen one after the other, using the relationships **immediately_preceded_by and immediately_precedes**.

**Figure 8:**
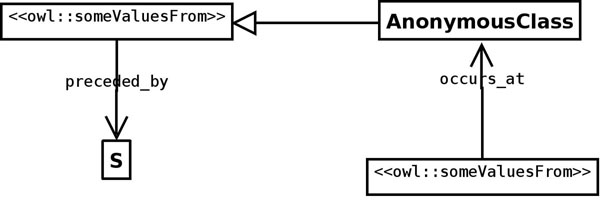
**UML diagram of a query that demonstrates the utility of the Sequence ODP.** The OWL-DL query **occurs_at some (preceded_by some S)** returns any interaction that occurs after S (G2 and M). However, if a user is only interested in anything occurring immediately after S (G2 but not M) **immediately_preceded_by** should be used: **occurs_at some (immediately_preceded_by some S)**.

**Figure 9:**
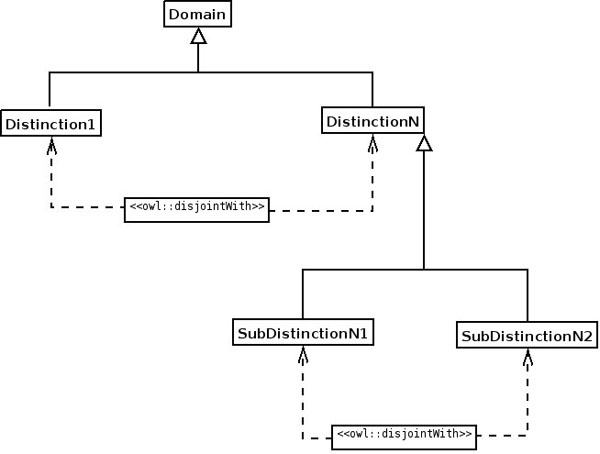
UML diagram of the Upper Level Ontology ODP

**Figure 10:**
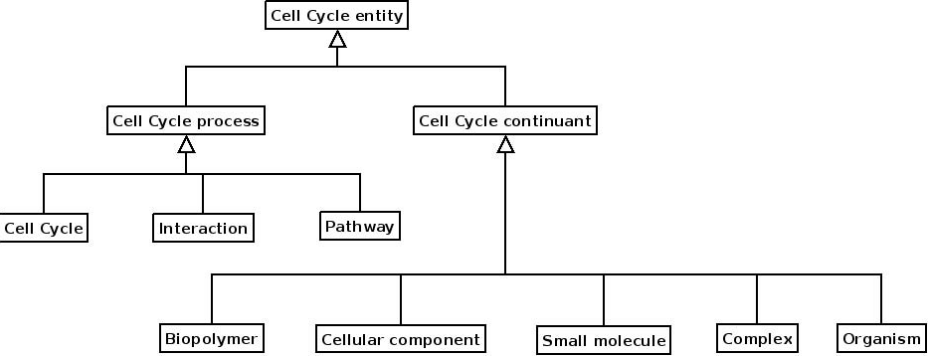
**UML diagram of the Upper Level Ontology ODP, as applied in CCO.** Disjoint axioms not shown.

• Extensional ODPs: ODPs that provide ways of extending the limits of the chosen KR language. Some ODPs can be used to overcome those limitations and present a suitable representation of the knowledge domain that needs to be captured. For example OWL cannot be used to express exceptions [7,17] or n-ary relationships [7], and there are ODPs to work around those limitations (Exception ODP [18] and N-ary Relationship ODP [19]).

• Good practice ODPs: ODPs that are used to ensure a modelling good practice. These ODPs are used to produce more modular, efficient and maintainable ontologies, tackling already known pitfalls of ontology engineering such as hard-coding of multiple subsumptions [20]. Examples include Normalisation ODP [21], Value Partition ODP [16] and Upper Level Ontology ODP [15].

• Domain Modelling ODPs: ODPs that are used to model a concrete part of the knowledge domain. They can be defined as “signature” ODPs: each knowledge domain has its peculiarities and these ODPs are used to model those peculiarities. Biological knowledge sometimes differs from other domains because of contingency, symmetry, different levels of complexity interacting with each other, emergent properties, *etc*. Examples include List ODP [22], Adapted SEP ODP [23], Sequence ODP [14] and Species ODP [24].

Extensional and Good Practice ODPs are common to all ontologies. Domain Modelling ODPs are more specific to the knowledge domain (in this case, biological knowledge), but they can also be used in other domains.

### Applying ODPs

An important aspect of ODPs is understanding when it is appropriate to apply a particular ODP. Ideally, the situation in which it is appropriate to apply an ODP should be apparent to a bio-ontologist; the ODP should be self explanatory in terms of documentation (for example when exploring the ODPs public catalogue presented bellow). The bio-ontologist can, however, be guided using competency questions such as the ones described in Table 1. These and other questions will be used to form a decision tree that will guide a bio-ontologist towards an appropriate ODP. These questions can be supplemented with material on the types of entity that can be involved and the kinds of relationships they have with other entities, eventually being refined down to the granularity of semantics used in the ODP itself.

**Table 1:** Examples of competency questions that help in choosing an appropriate ODP

**Question**	**ODP**
Do these values exhaust the space of possibilities for a value space?	Value Partition
Is this a structure where the order matters?	List
Are those features of a relationship?	N-ary Relationship
Does it constitute an exception of a default case?	Exception

Once chosen, the main method for applying an ODP is to recreate completely or in part the structure of the example ODP in the ontology, optionally reusing (“importing” in OWL parlance) parts of the example ODP. The user can be guided in the process with wizards, for example using the wizards provided by the CO-ODE project [25] for the Protégé ontology editor [26].

Another method of applying ODPs is to use condition matching. The Ontology Processing Language (OPL) is a syntax that allows conditions to be defined for matching classes in an ontology written in OWL. The classes matched have transformations applied on them that change axioms or annotation values. Thus OPL can be used to create ODPs in an ontology, by defining and ODP as the changes to be made when a match happens.

The matching condition can be of two types:

• Syntactic condition: the condition relies on a string value. Thus, the class name or any annotation value, such as **label** or **comment**, can be used to try to match the condition. The condition can be a given value (e.g. **cell differentiation**) or a regular expression (e.g. **(.+?) (differentiation)**).

• Semantic condition: the condition relies on the semantic structure of the ontology—the ODP is applied to any classes matching a class expression. For example, a condition can be defined so as to match any class that is subsumed by the class expression **located_in all (Chromosome or (part_of some Chromosome))** (in other words, any class that has the expression **located_in all (Chromosome or (part_of some Chromosome))** as a necessary condition). A semantic condition can be as complex as the user wishes (using any of OWL's expressivity) as the reasoner [27,28] will process the ontology and retrieve any matched classes.

OPL is partially based on the Manchester OWL Syntax [29] and SPARQL [30], and it is available as a standalone application [31] or as a Protégé plugin [32]. The OPL commands are written in a flat file by the user and the OPL program parses the file, selecting classes and applying the changes defined, creating a new ontology. ODPs can be codified in the defined changes, and human-readable explanations can be written in comments. Thus, the ODPs are stored in a flat file for direct application, together with any comments bio-ontologists might find of interest. Therefore, ODPs can be applied at any time, to any ontology, by running the OPL program, and are persistently stored (Figure 3).

### Documenting ODPs

The documentation system proposed in this research is inspired by the original SDPs documentation system [12], with some changes; the basic system is essentially the same, relying on some predetermined sections with which each ODP must be described; name, structure, *etc*. In the case of ODPs the sections are different, and some of them are optional (Table 2). An implementation of the documentation system is available as an ODPs public catalogue [13]. The catalogue is directly implemented in OWL: each ODP is described in an ontology, using annotation properties for the sections that describe the ODP. The semantics of the ODP are directly expressed in the ontology, allowing for importing the ODPs and sharing the ODPs together with all the information codified in the annotation properties. Each Ontology is translated to HTML by an script (OWL2HTML) and the URL of the ontology is automatically generated from the URI of the ontology. The whole catalogue can be downloaded [33] and generated locally from the OWL files by running the OWL2HTML script. The catalogue is open for suggestions and corrections, and any user can propose new ODPs to be added using the mailing lists and forums provided by Sourceforge [33].

**Table 2:** Documentation system sections and their explanation. The names of the sections are given in the left column; the explanation in the center column, and the right column states which sections are optional

**Section name**	**Explanation**	**Optional**
Name	The unique name of the ODP	No
Also known as	Any other name that is given to this ODP	Yes
URL	An URL where the ODP can be obtained	No
Classification	The classification, by general usage, of the ODP: One of “Extensional”, “Good practice” or “Domain Modelling”	No
Motivation	The scenario where the ODP might be needed	No
Aim	The concrete solution the ODP provides	No
Elements	The properties, classes and instances that build the ODP	No
Structure	How the elements relate to each other to build the ODP. This should be provided in any graphical form like UML or OWLViz	No
Sample	An example of the structure, applied to an ontology	No
Implementation	Explanation of how to build or apply the ODP in an actual system	No
Result	The structure that should appear in the ontology after applying the ODP (and often after reasoning)	No
Side effects	Any non obvious consequences of applying the ODP	No
Known uses	Any system where the ODP has been successfully applied	Yes
Related ODPs	Any ODP that uses or is used by this ODP, or any ODP that has anything in common with this one	Yes
References	Any publications or web pages where this ODP has been previously described	Yes
Additional information	Any information that does not fit in any of the previous sections	Yes

SDPs and ODPs are described in a different manner in a documentation system. SDPs are described with UML in a generic manner, and then the instances of the SDP are applied in the programming language of choice. In contrast, there is no easy, graphical, complete and established language a la UML for describing ODPs, because there is not such a language for KR languages. As a consequence, ODPs have to be described using instances: the model, rather than being a generic structure like in SDPs, is an instance that implicitly describes the generic structure.

The absence of a language that can describe the same structure in different KR languages makes it very difficult to develop a suitable graphical representation for ontologies. In the case of the ODPs public catalogue UML has been chosen in the hope that better languages will be developed. Despite having certain advantages (standard, already widely used and with available tooling) and having been designed as a general purpose modelling language, UML lacks native structures for a straightforward representation of OWL. Therefore, the UML representation is not compact enough and it is too complex. The UML to OWL mapping used in the public ODPs public catalogue is the one proposed in [34] (Figure 4). OWLViz [35] is also used for simple subsumption hierarchies.

### Use of ODPs on the Cell Cycle Ontology

In the context of the FP6 project DIAMONDS [36], an ontology is being developed to represent the knowledge about the cell cycle [37]. This application ontology, called Cell Cycle Ontology [38], comprises data from a number of resources such as GO, Relations Ontology (RO) [39], IntAct [40], NCBI taxonomy [41], UniProt [42] as well as data from DIAMONDS partners. The resulting CCO is designed to provide a richer view of the cell cycle regulatory process, in particular by accommodating the intrinsic dynamics of this process. For that purpose, three major components are considered: the (persistent) entity itself, its spatial localization, and its temporal localization. CCO provides a test bed for the development of new approaches and tools necessary to create a fully-fledged knowledge base. This knowledge base is expected to enable deployment of advanced reasoning approaches for knowledge discovery and hypotheses generation. CCO supports four model organisms: human (*Homo sapiens*), Arabidopsis (*Arabidopsis thaliana*), Baker's yeast (*Saccharomyces cerevisiae*) and Fission yeast (*Schizosaccharomyces pombe*). There is an ontology file for each of the four model organisms and the file is available in several formats [43]: OBO [4], OWL-DL [5], XML, DOT [44] and GML [45]. Presently, CCO holds more that 20,000 concepts (more than 1,000 bio-molecules and over 9,000 interactions) and more than 20 types of relationships. At present, two ODPs have been applied in CCO: the Sequence ODP and the Upper Level Ontology ODP.

The Sequence ODP [14] is used in CCO to model the cell cycle (Figure 5). The cell cycle is modelled as a sequence of events, starting in the phase G1, followed by S, G2 and finally M [46]. For the sake of simplicity, only the described steps of a standard cell cycle are considered, not considering the other steps (that also might play important roles) or variations such as endoreduplication.

There are other sequences of events that in principle can be modelled in the same manner, such as metabolic pathways. This ODP is a “trimmed down” version of another ODP, the List ODP [22]. The List ODP is a much more complex structure in which the exact order of items is very important, whereas in the Sequence ODP the only aspect modelled is what happens after or before a given event. For example, the Sequence ODP cannot be used to compare different sequences of events. The sequence ODP (Figure 6) makes use of the relationships **precedes** and **preceded_by** from RO, both being transitive. It also uses two relationships not present in RO, namely **immediately_precedes** (subproperty of **precedes**) and **immediately_preceded_by** (subproperty of **preceded_by**), both being functional.

When the ODP is applied to CCO (Figure 7), each phase of the cell cycle is **immediately_preceded_by** a phase and **immediately_precedes** another phase, only one in both cases, due to the fact that **immediately_preceded_by** and **immediately_precedes** are functional. Any phase that is **immediately_preceded_by** one phase is also assumed to be **preceded_by** the same phase, because **preceded_by** is a superproperty of **immediately_preceded_by**. The same applies to **immediately_precedes** and **precedes**.

The use of the Sequence ODP allows to do flexible queries against the ontology. For example, if a given interaction occurs at M, and a query is defined to retrieve anything that happens after S (Figure 8), a reasoner will retrieve the interaction (and any interaction occurring at G2, as both G2 and M are preceded by S). This is due to the transitivity of **preceded_by**, which is assumed to relate the pertinent phases by the reasoner (even if it is not explicitly stated in the ontology) because it is the superproperty of the actual property that has been used to assert the relationship in the model, **immediately-preceded_by**. However, if the user is only interested in something happening just after S (G2 but not M), **immediately_preceded_by** should be used instead.

The Upper Level Ontology ODP [15] (Figure 9) can be used to facilitate modelling through its basic ontological distinctions. A principle application of upper level ontologies is to integrate different ontologies. This can be done because an upper level ontology makes distinctions between classes, independent of any particular domain: the classes in it represent types of concepts, such as **physical entity**, **process**, *etc*. For example, if an ontology that describes processes needs to be integrated, it can be done so under the class **process**. The classes of the upper level ontology are generally created according to philosophical criteria such as continuants vs. occurrents. Therefore, the use of an upper level ontology is controversial, because there are many flavours of philosophical approach and the bio-ontologist may follow a particular view of the world that will highly influence the structure of the bio-ontology. In the case of CCO an upper level ontology has been created (Figure 10) to include classes from other ontologies such as the whole **Cell Cycle** subontology from GO. The use of an upper level ontology also helps to ensure a good modelling practice, as different kinds of classes (processes, molecules) are created in separate disjoint subtrees, resulting in a cleaner model.

## Discussion

**Figure 11:**
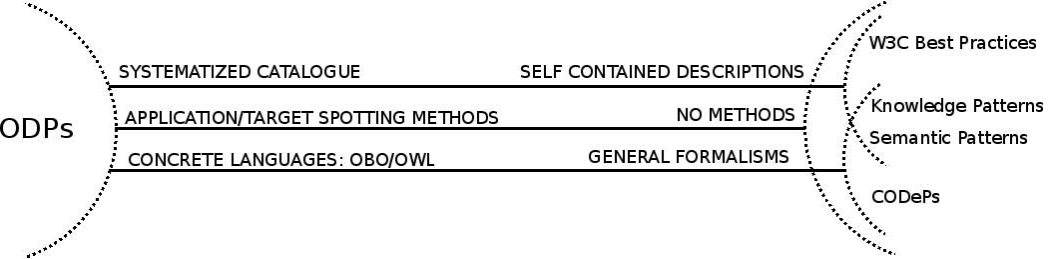
**Comparison of proposed ODPs (left) and previous work (right)**. Three criteria are used for comparison: application and target spotting methodologies, documentation and types of formalisms.

Figure 11 shows a simplified overview of prior attempts to provide solutions similar to ODPs; for the sake of brevity, the “W3C Best Practices” are assumed to be equivalent to the patterns described in [47-57] (see bellow).

Even if ODPs have already been documented in the literature, they have not been explicitly mentioned as such until recently [58-60]. In [60] they are mentioned as part of some ontology building methodologies, without further analysis such as documentation and application. The idea of CODePs (Conceptual Design Patterns) [58,59] is close to ODPs, but they differ in the level of granularity of the proposed solution; CODePs are necessarily less fine grained than ODPs, as they represent conceptual and general solutions, whereas ODPs offer solutions in a given KR language with full semantic coverage. We propose that CODePs and ODPs are complementary: a CODeP will incarnate itself in an ODP that will show the bio-ontologist how to implement the CODeP in a concrete KR language, much as happens with the CODeP Description-Reification and the N-ary Relationship ODP [19] in OWL. In summary, the application procedure, documentation system and representation of ODPs and CODePs are different. Knowledge Patterns [61] are conceptual general patterns that are “morphed” into a given knowledge base by a set of mapping axioms. Thus, the knowledge pattern can not be applied directly by the bio-ontologist: this is a drawback since the application of the pattern needs to be as intuitive as possible. The same argument applies for the Semantic Patterns [62]. The ODPs presented herein are real solutions to biological knowledge modelling problems, rather than theoretical propositions of general patterns; the value of these ODPs is that they are ready to be used by bio-ontologists, without any morphing axiom. ODPs are presented in OWL to make full use of the language's semantics. Those semantics can be mapped to other languages for interoperability (for example is relatively easy to map from OWL to OBO [63-66]), but the opposite does not often happen: it is difficult for bio-ontologists, given a pattern in an abstract formalism, to apply that pattern to an actual bio-ontology with a concrete KR language.

Some attempts have been made to provide best practice guidelines in ontology engineering and KR, which in some cases are semantically equivalent to ODPs. Some of those efforts have been collected (albeit not as a systematized collection) in the W3C Semantic Web Best Practices and Deployment Working Group web [67]. Other efforts have been published as self-contained patterns in single publications, regarding partonomy [47,48], transitive propagation [49-52,68], ontology level [53,54] and multi-ontology level best practices [55,56], and granularity [57], to mention some representative examples. In all cases, documentation, graphical representation and application methodologies as such have not been addressed in detail; at best, they were only implicitly and partially used. Some of those ODPs are collected in the ODPs public catalogue [13].

The use of ODPs will most likely give several advantages to bio-ontologists when creating and maintaining bio-ontologies. The following advantages have not been thoroughly tested, and therefore there is not experimental evidence for them, but they are reasonable assumptions based in the authors' experience in bio-ontology engineering. The advantages are divided in three areas:

1. Design (semantics, modelling):

• Rich and granular modelling. ODPs should facilitate the production of more richly axiomatised ontologies by allowing a more fine-grained modelling of the knowledge domain. They should help in making the implicit knowledge found, for example, in term names, explicit, encoding it in the semantics of the ontology. Additionally, bio-ontologies are deepening the knowledge they model, and ODPs to represent that deeper knowledge with the suitable granularity are needed.

• Semantic encapsulation. ODPs provide an easy way of dealing with the complexity of semantics in conceptual modelling by encapsulating it in the ODP.

• Robustness and modularity. Some ODPs help in creating more robust and modular ontologies.

• Reasoning. The richer axioms needed for efficient and productive reasoning should be reached more easily using ODPs. Therefore, as more axioms are placed in the ontology more sophisticated inferences can be undertaken.

• Alignment. More and more ontologies are being developed and efficient ways for comparing/aligning them are consequently necessary. The consistency of modelling inherent in the use of ODPs should support semantic matching between different ontologies.

2. Implementation (actual development of the ontology):

• Focused development. Having an ODP as an engineering artefact should reduce the development time, so that the domain expert can be focused on the modelling details of the specific area that is being modelled.

• Tooling. ODPs can be codified programmatically, providing tools that can automatically build sectors of an ontology that are complex or regular. The same tools could also guide the ontologist in the process of building ontologies.

• Rapid prototyping. ODPs are ideal for rapidly developing prototypes. Having prototypes should allow developers to discuss complete models of ontologies in early stages and hence make more sound ontologies. It should also allow faster development.

• Re-engineering. ODPs could be applied in the beginning of an ontology development process as well as during the life cycle of it, providing, for instance, valuable insights for refactoring some components which may hold an inconsistency or which may violate design principles.

3. Communication:

• Good communication. The use of ODPs should improve communication between ontology developers. The developers could easily recognize the different features of the ontology produced by the ODP, as it represents a well known and thoroughly documented abstraction.

• Documented modeling. When creating ontologies the process should be more precisely documented by simply mentioning which ODPs were used. As a result, the design decisions would become explicit.

• Comprehension of advances in KR. KR languages are evolving fast (for example OWL 1.1 [69]) and it is usually difficult to understand the new features of the languages: by providing ODPs it should become much easier, as ODPs are examples of how to use the new features.

## Conclusion

ODPs are ready-made solutions for tackling complex modelling issues when creating and maintaining bio-ontologies. Moreover, they provide a bridge to rich and rigorous modelling. They also offer advantages in design (rich and granular modelling; semantic encapsulation; robustness and modularity; reasoning; alignment), implementation (focused development; tooling; rapid prototyping; re-engineering) and communication (good communication; documented modelling; comprehension of advances in KR). Even if ODPs present all those advantages, it remains essential that bio-ontologists are provided with ontology building blocks and tools to easily create and manage ODPs. A Protégé plugin is foreseen as a supporting tool that will allow the creation and storage of ODPs by means of a graphical and user-friendly environment, and there is already a Protégé plugin for applying ODPs that uses the ODPs public catalogue presented [70]. ODPs might ideally follow a path similar to SDPs: first, they are discovered or identified, then they are comprehensibly tested, and finally, they become part of the language or system itself. Such a process cannot occur within ODPs (and OWL which is much less extensible) to the same extent but in an ideal situation the ODPs might well be perceived by the bio-ontologist as something that comes “for free” in the language or the tools for supporting the development of ontologies.

In order for the ODPs to be used, not only tooling but also proper documentation is vital, and there are open issues in the documentation system presented. The most important problem is the lack of a proper graphical language for representing the structure of the ODPs; UML is acceptable for the current implementation but a better representation is needed and GrOWL [71] is a promising possibility. On the other hand, other UML to OWL mappings can also be used [58,72]. The sections for the documentation system might evolve in the future as ODPs are more widely used and identified, depending on the users' feedback. Two new sections are foreseen in the short term: a section with the questions for choosing ODPs (as in Table 1) and a section for the version of the ODP, including a backwards-compatibility mechanism. Classification of ODPs might well also evolve towards a classification adapted to the needs faced while building and maintaining ontologies. In the long term, metrics for evaluating ODPs will also be studied so that a degree of complexity will be associated to each ODP (in a section of the documentation system), that in turn will provide users more information about the ODPs they would like to use. The same metrics will also provide a way to measure the complexity of the resulting ontology.

The ODPs identified in CCO will support its sound evolution by being part of the CCO maintenance life cycle. There are still some ODPs that have not been completely explored such as ODPs dealing with temporal aspects or those dealing with negative (complementary) knowledge. Besides, many more ODPs within CCO and other bio-ontologies will be identified and added to the ODPs public catalogue. There are plenty of areas of biological knowledge that have not yet been explored to find possible ODPs, such as phylogeny, molecular interactions, *etc*. In any of those areas of biological knowledge the identification of ODPs will facilitate the development of rich and rigorous bio-ontologies. This will ultimately provide a robust and fine grained representation of the knowledge in biology, allowing for a more efficient knowledge management in the field, and increased exploitation of computational reasoning.

## Abbreviations

CCO: Cell Cycle Ontology.

CODeP: Conceptual Design Pattern.

GML: Graph Modelling Language.

GO: Gene Ontology.

HTML: Hypertext Markup Language.

KR: Knowledge Representation.

OBO: Open Biomedical Ontologies.

ODP: Ontology Design Pattern.

OOP: Object Oriented Programming.

OPL: Ontology Processing Language.

OWL: Web Ontology Language.

RO: Relations Ontology.

SDP: Software Design Pattern.

UML: Unified Modelling Language.

URI: Uniform Resource Identifier.

URL: Uniform Resource Locator.

W3C: World Wide Web Consortium.

XML: eXtensible Markup Language.

## Competing interests

The authors declare that they have no competing interests.

## Authors' contributions

MEA led this effort, working in the core ideas about ODPs and in the implementations (OPL and the ODPs public catalogue). RS provided expertise in KR and in general aspects of ODPs. EA provided expertise in the Cell Cycle Ontology and in technical aspects of ODPs. MK provided general ideas related to the Cell Cycle Ontology knowledge management system. All authors contributed to, read and approved the final manuscript.

## Acknowledgements

This work was supported by the EU (FP6, contract number LSHG-CT-2004-512143 to EA) and MEA received funding from MC-EST (VIB), EPSRC and Manchester University.

This article has been published as part of *BMC Bioinformatics* Volume 9 Supplement 5, 2008: Proceedings of the 10th Bio-Ontologies Special Interest Group Workshop 2007. Ten years past and looking to the future. The full contents of the supplement are available online at .
